# Investigation of the Use of Naturally Grown CaCO_3_ Crystals on Rocks as a Radiation Dosimeter via Thermoluminescence Method

**DOI:** 10.1002/bio.70217

**Published:** 2025-06-04

**Authors:** Huseyin Toktamis, Tamer Sertaç Güneş, Dilek Toktamis

**Affiliations:** ^1^ Department of Engineering Physics University of Gaziantep Gaziantep Turkey

**Keywords:** CaCO_3_, natural crystals, radiation dosimeter, thermoluminescence

## Abstract

This study investigates the thermoluminescence (TL) characteristics of naturally occurring CaCO_3_ crystals found on rocks collected from the Akçatekir region of Adana, Turkey. The research evaluates some of the radiation dosimeter parameters such as dose–response, heating rate, reusability, and fading. Both crystals demonstrate favorable TL properties with distinct TL glow curves. One of the crystals exhibits two prominent TL peaks at approximately 90°C and 235°C, while the other shows peaks around 115°C and 300°C. Both crystals display a broad linear dose–response range from 2 to 576 Gy. Although repeated experiments lead to a reduction in the low‐temperature peak, the dosimetric peak (near 220°C) remains largely unaffected. Additionally, TL intensity decreases as the waiting time between irradiation and measurement increases. However, the fading process does not compromise the structural integrity of the crystals or introduce new TL peaks. Given their wide linear dose–response range, strong reusability with standard deviation of 2.71%, and stable dosimetric peak under ambient conditions, these crystals appear to be promising candidates for dosimetric applications.

## Graphical abstract

This study investigates the thermoluminescence (TL) characteristics of naturally occurring crystals found on rocks. The research evaluates some of the radiation dosimeter parameters such as radiation dose, heating rate, reusability, and fading. The crystals demonstrate favorable TL properties and appear to be promising candidates for dosimetric applications.

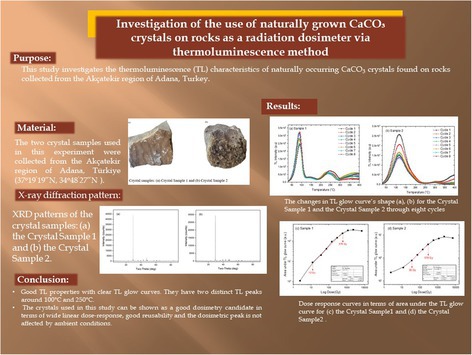

## 1 Introduction

Thermoluminescence (TL) is a phenomenon where a material emits light when heated after being exposed to ionizing radiation, and it plays a significant role in fields such as radiation dosimetry, archeological dating, and environmental monitoring [1, 2]. TL occurs as the stored energy in the material is released upon heating, making it a useful tool for accurately measuring the amount of ionizing radiation. TL depends on the material's structure and the environmental conditions it has been exposed to [3].

Crystals are solid materials with atoms, ions, or molecules arranged in a highly ordered, repeating structure. They form through crystallization, which occurs under natural conditions like magma cooling, precipitation from solutions, or sublimation. Factors such as temperature, pressure, and solvents influence crystal morphology and properties, affecting size, habit, and purity. These variations are essential for applications in pharmaceuticals and materials science [4, 5].

Naturally grown crystals are formed through various geological processes, each influenced by specific environmental conditions such as temperature, pressure, and chemical composition. The crystallization process plays a crucial role in determining the structural and TL properties of these crystals. For instance, magmatic crystals crystallize from molten rock as it cools, while hydrothermal crystals emerge from mineral‐rich fluids that fill rock cavities. Sedimentary crystals, on the other hand, precipitate from solutions, often influenced by evaporation or changes in temperature and pressure [6]. The distinct conditions under which these crystals form lead to variations in their atomic arrangements within the crystal lattice, which in turn affects their TL behavior [7].

The thermoluminescence properties of crystals are particularly significant in scientific and industrial applications. TL materials store energy from radiation in their crystal lattices, primarily through the trapping of free electrons and holes caused by lattice defects [7]. This stored energy is released as light when the material is heated, making TL properties useful for dosimetry and radiation detection. For example, manganese‐doped YAlO_3_ crystals have been shown to possess enhanced TL properties, making them suitable for applications in holographic recording and optical data storage, as well as for TL dosimetry [8]. Furthermore, the crystallization process can enhance the TL response, as evidenced by studies comparing the TL characteristics of doped and undoped glass‐ceramics, which demonstrated that crystallization significantly increases the thermoluminescence response [9].

Crystals that naturally grow on rocks, often referred to as mineral crystals, are significant in various fields, including geology, materials science, and optoelectronics. These crystals form through geological processes and can exhibit a range of optical properties that are influenced by their composition, structure, and the conditions under which they crystallize.

The formation processes of naturally grown crystals, including magmatic, hydrothermal, and sedimentary origins, significantly influence their structural and TL properties. These properties are critical for various applications, including environmental monitoring and geological dating, where accurate measurements of radiation exposure are essential. The interplay between crystallization conditions and TL behavior underscores the importance of understanding the geological processes that lead to crystal formation.

Magmatic crystallization is a fundamental geological process that occurs when molten rock, or magma, cools and solidifies, leading to the formation of various minerals. This process predominantly takes place deep within the Earth's crust, where high temperatures facilitate the crystallization of minerals as the magma cools. The cooling rate is a critical factor in this process; slower cooling allows for the growth of larger, well‐formed crystals, which are characteristic of igneous rocks. For instance, minerals such as quartz, feldspar, and olivine crystallize at different temperatures based on their unique chemical compositions, resulting in a diverse array of mineral textures and structures within igneous formations [10].

Additionally, the crystallization process is influenced by the presence of other minerals and the overall geochemical environment of the magma. For example, the study of clinopyroxene phenocrysts in Paleoproterozoic picrobasalts reveals that the evolution of magmatic systems is complex, involving interactions between different mineral phases and the surrounding crustal material. This complexity is further illustrated by the classification of pyroxenes into autocrysts, antecrysts, and xenocrysts, each reflecting different stages of crystallization and magma evolution [10]. The textural features of these minerals provide insights into the history of the magmatic system and the conditions under which crystallization occurred.

The TL properties of natural crystals are influenced by several factors, including the type of crystal, the nature of defects within the crystal lattice, and the thermal treatment applied to the samples.

Natural minerals such as quartz and feldspar are well‐known for their TL properties. For instance, studies have shown that quartz grains exhibit distinct TL glow curves that correlate with their irradiation history, with specific peaks at temperatures such as 260°C, 310°C, and 365°C, which correspond to different trap activation energies [11]. The glow curves of these minerals can shift to higher temperatures with increased burial depth and environmental temperature, indicating their potential use in geological dating [12]. Furthermore, the TL characteristics of calcite have been modeled using machine learning techniques, demonstrating the complexity and variability of TL responses in different mineral types [13].

Many researchers have investigated the TL properties of natural crystals. Among these studies, Doğan and colleagues [14] examined the TL characteristics of natural onyx from Turkey. Their research involved beta irradiation (^90^Sr/^90^Y) of onyx samples, followed by computerized glow curve deconvolution (CGCD) analysis, which identified six distinct peaks. The onyx samples exhibited TL glow peaks at different temperatures (150°C, 240°C, and 325°C) when irradiated with doses ranging from 2.4Gy to 2.457kGy. Notably, the high‐temperature peaks demonstrated a linear dose–response, indicating potential suitability for high‐dose dosimetry. Additionally, they found that increasing the heating rate led to a decrease in TL peak intensities, highlighting the effect of heating on TL peak temperatures. This study suggests that natural onyx could be a viable candidate for dosimetry applications in high‐dose settings.

In another study, Yazici and colleagues [15] on the thermoluminescent dosimetric characteristics of rubidium chloride (RbCl) single crystals doped with europium (Eu) and hydroxyl (OH) ions. They analyzed the dose–response, signal fading, and reusability of these crystals, noting that the TL response was significantly influenced by dopant concentration and thermal treatment prior to irradiation. Optimal dosimetric properties were observed at 0.1 mol% OH and 1.5 mol% Eu, with a pronounced glow peak around 215°C, which made the material suitable for high‐dose applications ranging from 0.01 to 300Gy in medical and industrial environments. However, the study also indicated that further research is needed to enhance sensitivity relative to commercially available dosimeters, such as TLD‐100.

Studies on the TL properties of naturally grown crystals have shown that these materials provide valuable insights into Earth's history and geological processes. The structure and TL behavior of these crystals are closely related to their mineral composition and the environmental factors during their formation. However, literature on the TL properties of naturally grown crystals remains limited, with few comprehensive studies addressing their potential.

This study investigates the TL characteristics of naturally occurring CaCO_3_ crystals found on rocks collected from the Akçatekir region of Adana, Turkey. Various studies [16, 17, 18, 19, 20] have explored the TL characteristics of different types of calcite using a range of radiation sources. Overall, these investigations found that calcite displays TL behavior marked by three separate glow peaks, typically occurring within the temperature ranges of 100°C–150°C, 200°C–300°C, and 310°C–400°C. These peaks also demonstrate a strong linear correlation between TL intensity and radiation dose.

TL characteristics of calcium carbonate (CaCO₃) have gained increasing interest in recent scientific research due to their relevance in fields such as radiation dosimetry, archeological age estimation, and geoscience. This review consolidates key discoveries regarding the TL behavior of calcium carbonate—particularly calcite—and explores its interdisciplinary significance. A notable contribution by Kalita and Chithambo [21] analyzed both TL and infrared‐stimulated luminescence in limestone, a natural variant of CaCO₃. Their study revealed distinctive glow curves that are essential for dosimetric use, with specific temperature peaks and luminescent features linked to the material's crystal structure. These results support the use of TL in radiation detection applications involving natural carbonates.

In a more specialized study, Toktamış et al. [22] focused on calcite formed through microbial activity in organic‐rich soils. They found that the TL signals are highly sensitive to environmental conditions during formation and later geological changes. The complex glow curve patterns reflect the diverse nature of trap centers, underscoring the impact of biological and geochemical processes on TL behavior. Şahiner et al. [23] expanded on the dating potential of CaCO₃ by evaluating the shortcomings of optically stimulated luminescence (OSL) in these materials. Their findings suggest that TL techniques offer valuable insights for dating, especially in cases involving biogenic calcite, which often retains unique radiation exposure histories that TL analysis can help reconstruct.

Meanwhile, Işık et al. [17] introduced a novel approach by applying machine learning—specifically, long short‐term memory (LSTM) neural networks—to analyze TL data from CaCO₃. Their work demonstrated that factors like fading, heating rate, and measurement repetition significantly affect TL properties. This modern analytical method enhances the ability to interpret and predict luminescent behavior in such materials.

The motivation behind this research is to thoroughly explore the TL properties of naturally grown crystals. These crystals are known to have great potential, especially in fields such as radiation dosimetry and material science [24]. In this study, naturally grown CaCO₃ crystals on rock collected from the Akçatekir region of Adana, Türkiye, were analyzed to investigate their TL behaviors under beta source. The study focuses on the TL glow curves, peak intensities, peak temperatures, and the overall TL response of these crystals.

The key research questions include: How do different beta radiation doses, cycle of measurement, different heating rate and different storage time in a dark room affect the TL properties of these crystals? To answer these questions, detailed experiments such as the dose response experiment, reusability experiment, heating rate experiment, and fading experiment will be conducted.

## 2 Material and Experimental Preparations

### 2.1 Materials

**FIGURE 1:**
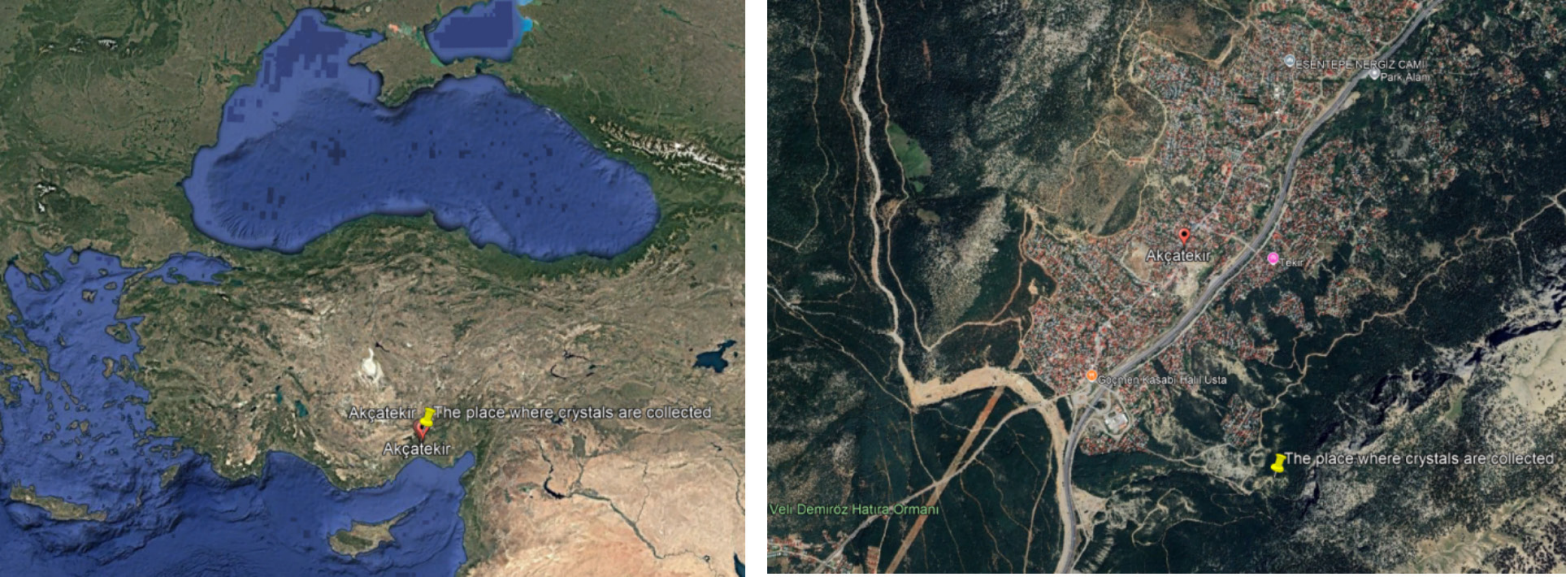
The location where the crystals are collected.

**FIGURE 2:**
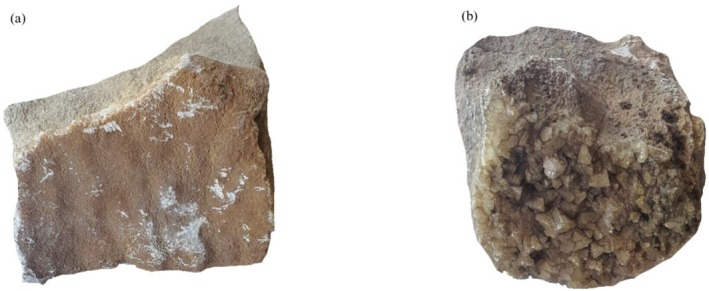
Crystal samples: (a) Crystal Sample 1 and (b) Crystal Sample 2.

sThe two crystal samples used in this experiment were collected from the Akçatekir region of Adana, Türkiye (37°19′19″N, 34°48′27″N). The location where crystals are collected and their raw pictures were shown in Figures 1 and 2, respectively.

**FIGURE 3:**
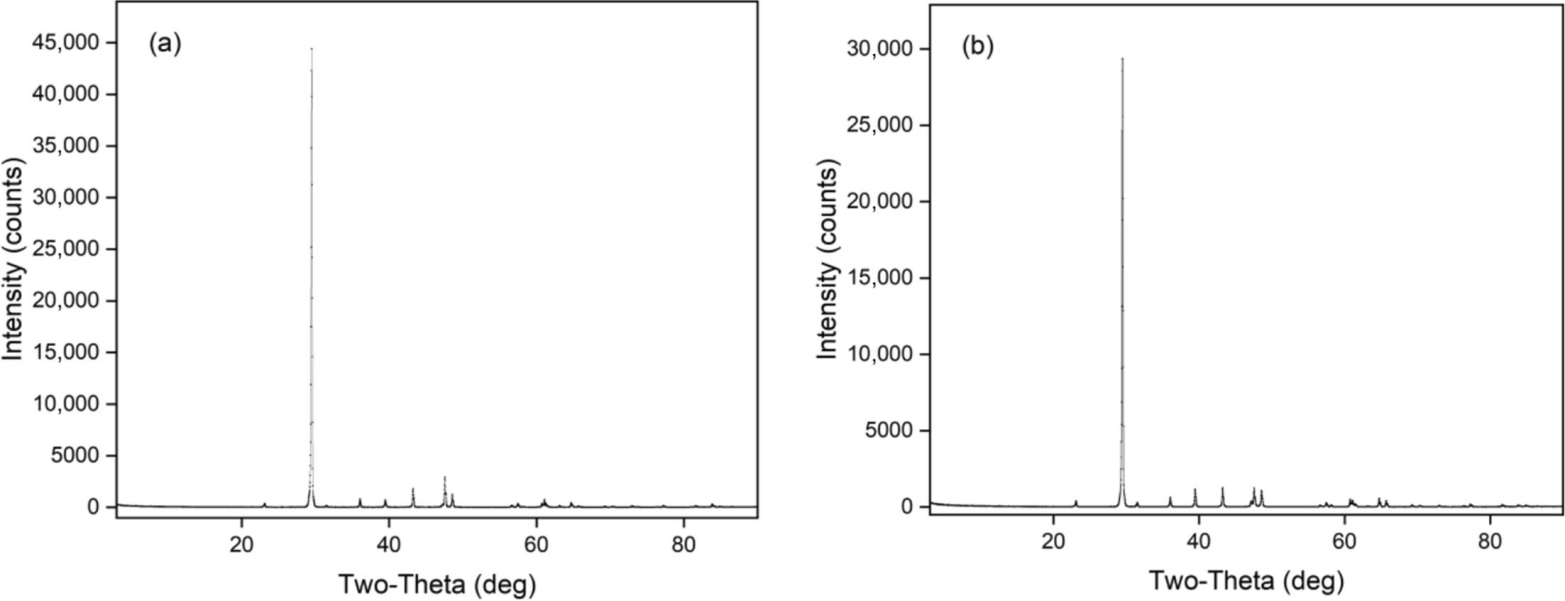
XRD patterns of the crystal samples: (a) Crystal Sample 1 and (b) Crystal Sample 2.

The XRD patterns of the crystal samples are presented in Figure 3.

The X‐ray diffraction (XRD) patterns and the extended peak identification reports for both crystals were obtained using a Rigaku D/MAX‐Ultima+/PC X‐ray diffractometer, available at the XRD Facility of Bogaziçi University's Advanced Technologies R&D Center. The results are presented in Figure 3 and Tables 1 and 2. This diffractometer is designed for versatile applications. Its X‐ray generator operates within a voltage range of 20–60 kV, a current range of 2–80 mA, and a maximum output of 3 kW, with SCR phase control. The generator has a focal spot size of 1 × 10 mm^2^.

**TABLE 1:** Extended peak identification reports for Crystal Sample 1.

2*θ*	*d* (Å)	Height %	Phase ID	hkl
29.479	3.0275	100.0	CaCO_3_	(0 1 2)
43.238	2.0907	4.1	CaCO_3_	(2 0 2)
61.081	1.5159	1.7	CaCO_3_	(2 0 8)

**TABLE 2:** Extended peak identification reports for Crystal Sample 2.

2*θ*	*d* (Å)	Height %	Phase ID	hkl
29.478	3.0276	100.0	CaCO_3_	(1 0 4)
43.222	2.0914	4.3	CaCO_3_	(2 0 2)
64.721	1.4391	1.9	CaCO_3_	(3 0 0)

Extended peak identification reports of the crystals in Tables 1 and 2 show that both crystals are calcite (CaCO_3_) based.

### 2.2 Preparation of the Samples

After the samples were collected, they were carefully washed and dried at room temperature. The crystal samples were then finely crushed and ground using a mortar to obtain a uniform powder. Subsequently, the samples were washed with a dilute hydrochloric acid (HCl) solution to remove any organic residues and potential surface impurities. Following the acid treatment, the samples were thoroughly rinsed several times with deionized water to ensure the complete removal of any remaining HCl.

After rinsing, the samples were dried once again at room temperature to avoid thermal degradation. For the experimental procedures, the aliquots of 15 mg were meticulously prepared from each crystal samples, ensuring consistency across all measurements.

### 2.3 Equipments Used in the Experiments

An irradiator device is specialized equipment designed to expose samples to beta particles from a controlled radiation source, such as the ^90^Sr‐^90^Y beta source. This device plays a critical role in scientific experiments where precise and regulated radiation exposure is required, including studies in dosimetry and other radiation‐related research fields. By allowing researchers to accurately control both the radiation dose and the exposure duration, it facilitates the simulation of specific radiation conditions and enables the observation of their effects on the samples. They were irradiated at room temperature using a ^90^Sr‐^90^Y beta radiation source with an activity of approximately 2.12 GBq (57 mCi). While the beta radiation source delivers 0.040 Gy/s, the distance between the radiation source and the sample was set at 1 cm.

The Harshaw TLD System 3500 is a device used for thermoluminescent dosimetry (TLD) measurements. It is specifically designed to measure the radiation dose absorbed by samples that have been exposed to radiation. When the samples are heated, the device releases the stored energy in the form of light. The intensity of this emitted light is proportional to the radiation dose absorbed by the sample. The measured light is detected and quantified by the system, and the data is transferred to a connected computer for analysis. All radiation dose information is automatically recorded and stored in the computer, ensuring accurate and reliable data management for subsequent research and analysis.

## 3 Experimental Results and Discussion

Four experimental approaches were employed to evaluate the TL properties of the naturally grown CaCO₃ crystals studied here: dose response, reusability, heating rate, and fading experiments.

### 3.1 Dose Response

The dose response experiment is conducted to evaluate the TL response of a material when exposed to different radiation doses. In this experiment, Crystal Sample 1 and Crystal Sample 2 were subjected to beta radiation doses ranging from approximately 2Gy to 7kGy. As soon as the irradiation was finished, the samples were heated from $30^{{}^{\circ}}C$ to $400^{{}^{\circ}}C$ in $1^{{}^{\circ}}C/s$ increments by the TLD reader. The primary objective of the experiment was to analyze the TL response of the crystals at various dose levels.

**FIGURE 4:**
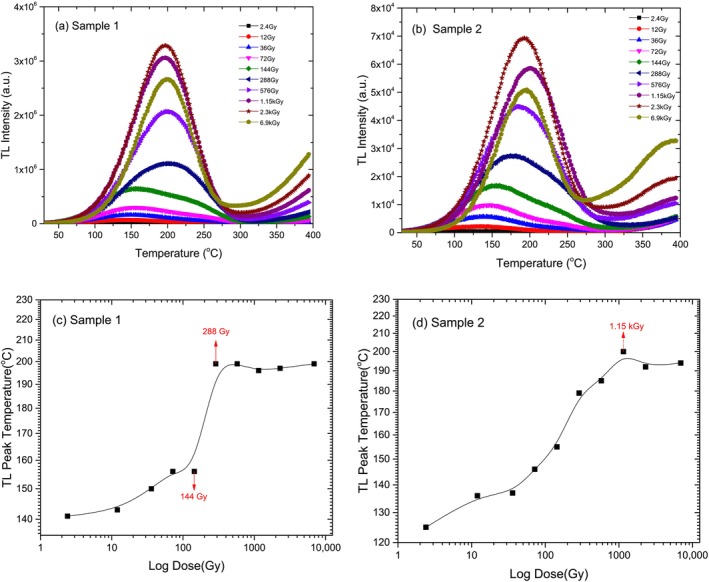
TL glow curves and peak temperatures of (a, c) Crystal Sample 1 and (b, d) Crystal Sample 2 at different beta radiation doses.

Figure 4a,b shows the changes in TL glow curves, and Figure 4c,d shows the changes in TL peak temperatures for Crystal Sample 1 and Crystal Sample 2 after exposure to different beta radiation doses. The glow curves of both samples up to 300°C exhibit a complex structure, consisting of at least two TL peaks with maxima near 150°C and 250°C. As the radiation dose increases, the overlap between these peaks becomes more pronounced, giving the impression of a single broadened peak. Consequently, the figures that depict a shift in the peak maximum with increasing dose are misleading and incorrect, as they do not account for the composite nature of the TL signal. Most of the observations described in Crystal Sample 1 were also detected in Crystal Sample 2, as shown in Figure 4b,d.

There has been no change in the number of TL peaks for either Crystal Sample 1 or Crystal Sample 2. Overall, no significant alterations in the TL glow curve shapes were observed with changes in radiation dose. At higher doses, both crystals exhibit a more distinct and sharp TL glow curve shape. When the crystals are compared simultaneously, the TL glow curve of Crystal Sample 1 appears more stable and consistent compared to that of Crystal Sample 2.

Figure 4c,d provides important insights into the differences in energy storage dynamics and trap depths within the crystals as a function of dose. Both Crystal Sample 1 and Crystal Sample 2 exhibit an increase in TL peak temperature with radiation dose, followed by stabilization at higher doses. However, Crystal Sample 1 reaches saturation at a lower dose (288 Gy), while Crystal Sample 2 continues to show changes up to 1.15 kGy before stabilizing. This suggests that the trap depth variations in Crystal Sample 2 extend over a broader dose range, whereas Crystal Sample 1 reaches its saturation point earlier. The shift of the TL peak temperature to higher temperature regions with increasing dose means that the trap depth of the respective trap shifts deeper.

The variation in TL intensity provides insight into the efficiency with which each crystal absorbs and stores energy, as well as how this energy is released upon heating. By analyzing the TL intensity at different radiation levels, it is possible to determine the relationship between the dose and the amount of energy trapped within the crystal lattice, and how this energy is released as luminescence during the heating process.

**FIGURE 5:**
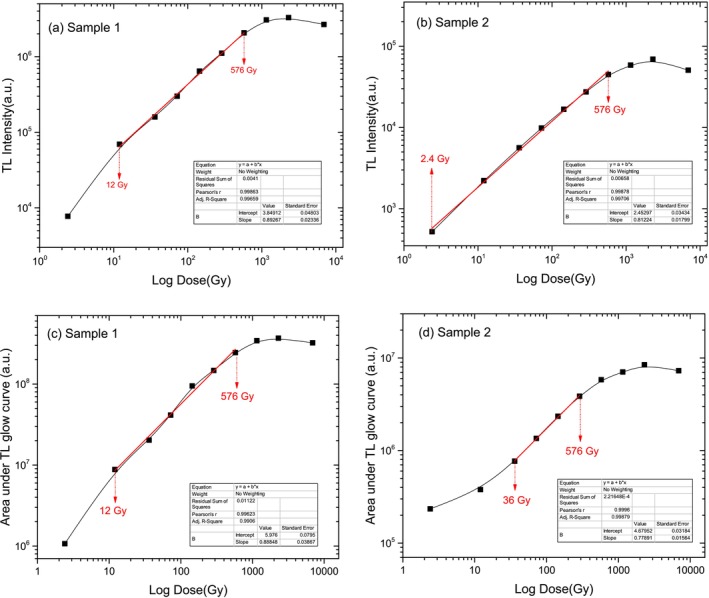
Dose response curves in terms of TL intensity and area under the TL glow curve for (a, c) Crystal Sample 1 and (b, d) Crystal Sample 2.

The graphs given in Figure 5a,b show the variation of TL peak intensity of Crystal Sample 1 and Crystal Sample 2 to varying beta radiation doses.

As shown in Figure 5a,b, both crystal samples exhibit a linear increase in TL intensity up to 576 Gy, after which the intensity reaches saturation. This indicates that while the crystals efficiently store energy within this dose range, their capacity becomes limited beyond 576 Gy, preventing further increases in TL signal.

The area under the TL glow curve represents the total amount of energy released from the crystal during the heating process, and it is directly related to the number of trapped charge carriers within the material. The graphs, presented below in Figure 5c,d, show how the area under the TL glow curve changes for Crystal Sample 1 and Crystal Sample 2 as a function of different beta radiation doses.

Both the crystal samples show an increase in the area under the TL curve with dose, eventually reaching saturation. Crystal Sample 1 exhibits a broader linear response range (12 to 576 Gy) before saturation, while Crystal Sample 2 reaches saturation at a higher dose (around 2.3 kGy). These differences suggest variations in the structural properties or trapping mechanisms of the crystals.

### 3.2 Reusability

The crystal samples can show different TL responses each time they are measured in a TLD reader, and this variation may differ depending on the type of TL material. A good dosimeter is expected to provide consistent TL results in every reading. To understand this, a reusability experiment is performed. Reusability (cycle of measurement) is an important parameterand a suitable dosimeter should be used repeatedly and the sameresult obtained each time [25].

In this experiment each sample was irradiated about 72Gy using a ^90^Sr‐^90^Y beta source. Immediately after irradiation, the samples were transferred to the Harshaw TLD System 3500 and heated from 30°C to 400°C, with a 1°C/s, while TL readings were continuously recorded. This procedure was repeated consecutively for eight cycles for each crystal without any interruptions between the cycles to assess the stability and reproducibility of the TL response. The resulting data were analyzed to determine the consistency of the TL response and the potential impact of repeated irradiation on the crystal's luminescence properties.

**FIGURE 6:**
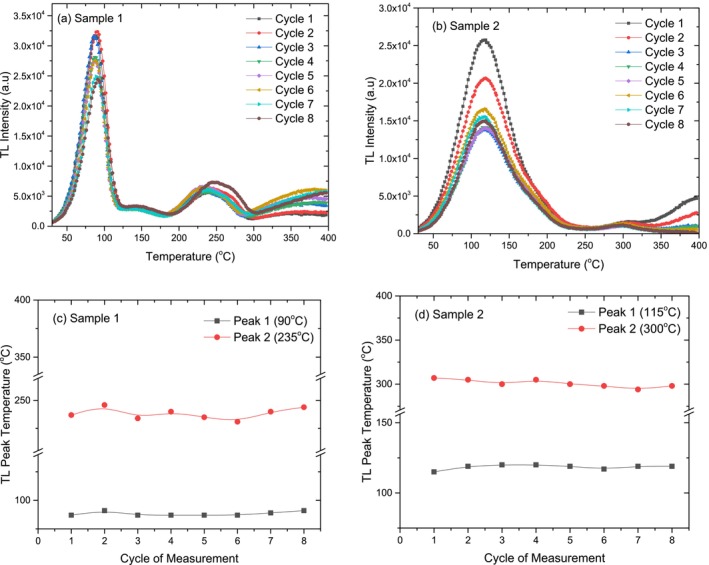
The changes in TL glow curve's shape (a, b) and TL peak temperatures (c, d) for Crystal Sample 1 and Crystal Sample 2 through eight cycles.

The graphs, presented in Figure 6, illustrate the changes in TL glow curve's shape and TL peak temperatures for Crystal Sample 1 and Crystal Sample 2 through eight cycles.

According to the results of Figure 6a,b, both the crystal samples maintain a consistent TL glow curve shape across repeated experiments, with no new peaks appearing or disappearing. In addition to these, both the crystal samples exhibit two distinct and stable TL peaks, with no significant changes in peak temperatures across eight cycles. This stability indicates that the trap positions remain unchanged, ensuring a consistent TL response through repeated measurements.

**FIGURE 7:**
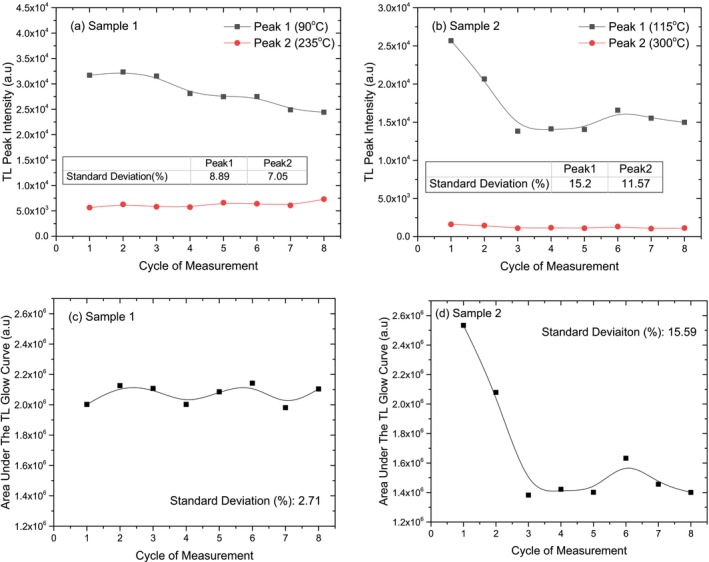
Variation of TL peak intensity and area under the TL glow curve as a function of measurement cycle for Crystal Sample 1 (a, c) and Crystal Sample 2 (b, d).

In the reusability experiment, the variation in TL peak intensity and area under the glow curve were analyzed for Crystal Sample 1 and Crystal Sample 2 over eight consecutive measurement cycles shown in Figure 7.

Figure 7a,b shows that both the crystal samples exhibit two distinct TL peaks, with deeper energy traps remaining stable while low‐temperature peak intensities decrease over repeated cycles. Crystal Sample 1 shows a continuous decline in low‐temperature peak intensity, whereas Crystal Sample 2 stabilizes after the third repetition. This suggests that shallow traps lose effectiveness over time, while deeper traps retain their stability.

The total area under the TL curve, as shown in Figure 7c,d, provides another layer of insight into how the TL response of both the crystal samples evolves over the course of multiple cycles.

As seen in Figure 7c,d, Crystal Sample 1 exhibits greater stability, with only minor fluctuations in the total area under the TL curve across cycles. In contrast, Crystal Sample 2 experiences a significant 40% decrease by the third cycle, followed by small fluctuations and partial recovery, though the overall trend remains downward. With reference to the area under the TL glow curve, the standard deviation values for Crystal Sample 1 and Crystal Sample 2 were calculated as 2.71% and 15.59%, respectively. This indicates that Crystal Sample 2 loses energy more rapidly, making its TL response less consistent compared to the more stable behavior of Crystal Sample 1.

### 3.3 Heating Rate

The heating rate experiment is designed to analyze how the TL properties of the samples are affected by reading at different heating rates. The primary aim of this experiment is to determine the influence of the heating rate on the TL glow curve's shape, TL peak temperature, TL peak intensity, and the total area under the TL curve, which represents the overall energy release.

In this experiment, the samples were first exposed to beta radiation for 72Gy. Following irradiation, they underwent the heating process. The experiment was conducted with four 15 mg samples, with heating rates of 1°C/s, 2°C/s, 3°C/s, and 4°C/s.

**FIGURE 8:**
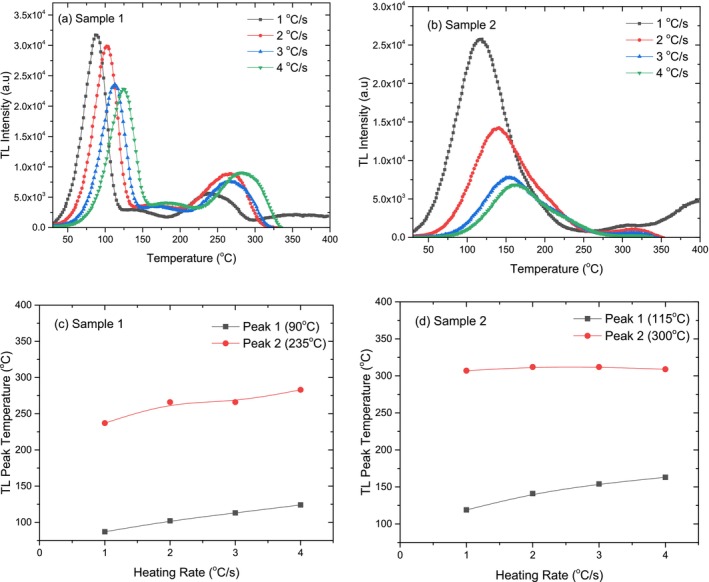
Variations of TL glow curves and TL peak temperatures at different heating rates for Crystal Sample 1 (a, c) and Crystal Sample 2 (b, d).

The TL glow curves obtained at different heating rates are shown in Figure 8a,b for two crystal samples. The effects of varying heating rates on the TL glow curve shapes were analyzed. For both the crystal samples, the results indicate that as the heating rate increases, no important change in the TL glow curve shape is observed. That means no extra peak is created or destroyed.

The effect of different heating rates on the TL peak temperatures for both crystal samples is depicted in Figure 8c,d. The low‐temperature TL peaks of both crystal samples shift 30°C higher with increasing heating rate. While the intermediate TL peak of Crystal Sample 1 shifts by 50°C, the intermediate peak of Crystal Sample 2 remains unchanged. This behavior is likely due to temperature lag caused by thermal contact between the heater and the sample, as well as the influence of sample thickness on temperature distribution at higher heating rates [26].

**FIGURE 9:**
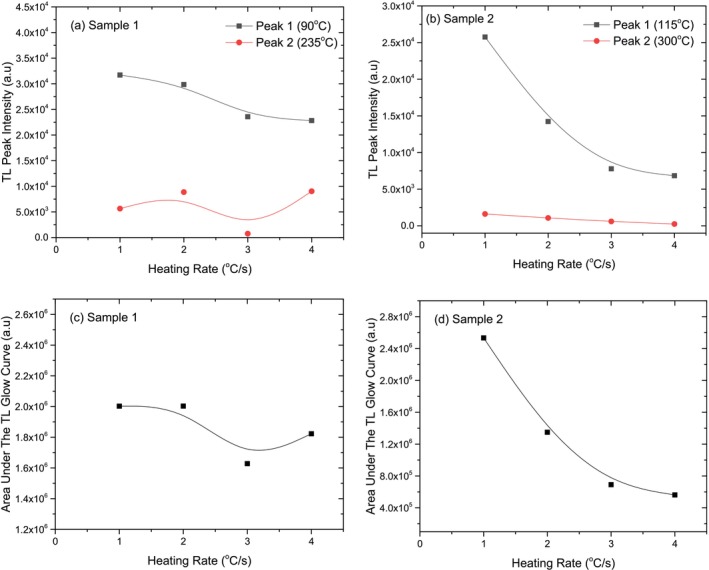
Variations of TL peak intensity (a, b) and area under the TL glow curve (c, d) as a function of heating rate for Crystal Sample 1 and Crystal Sample 2.

Figure 9 illustrates how the TL peak intensities and peak temperature changes across different heating rates for both crystal samples. As seen in Figure 9a,b, the TL intensity of the low‐temperature peak decreases as the heating rate increases, with Crystal Sample 2 experiencing a more significant reduction (60%) compared to Crystal Sample 1 (30%) after 4°C/s. While the intermediate peak of Crystal Sample 1 shows minor fluctuations, the intermediate peak of Crystal Sample 2 exhibits a slight decrease in intensity. This suggests that Crystal Sample 2 is more sensitive to heating rate variations, particularly at lower temperatures.

The changes in the area under the TL curve for both crystal samples at different heating rates are shown side by side in Figure 9c,d. The area under the TL glow curve of Crystal Sample 2 decreases significantly with increasing heating rate compared to Crystal Sample 1. By the end of a heating rate of 4°C/s, this decrease is recorded as approximately 10% for Crystal Sample 1, while for Crystal Sample 2, it is as high as 75%. Nonradiative transitions between centers can be a reason for the fall in TL intensity with increase in heating rate. Thermal quenching effect can be another reason for this reduction, as efficiency increases as temperature increases [27, 28].

### 3.4 Fading

The fading experiment was conducted to examine the natural decay of TL signals in the crystal samples over time. Fading refers to the gradual loss of stored energy in a material after exposure to radiation, without any external stimulation [29]. This process is crucial for understanding the stability of TL signals in applications such as dosimetry and archeological dating [30].

One of the primary reasons for fading is related to the quantum mechanical tunneling effects, which have been highlighted in several studies. For instance, Kitis et al. [31] discuss “anomalous fading” (AF), which refers to the unexpected rapid decay of high‐temperature TL glow peaks at room temperature, contrary to expectations from standard TL kinetic models. They attribute this rapid decay to a temperature‐independent tunneling effect, allowing trapped charges to escape from their traps without thermal activation. Another factor contributing to fading is the leakage of charged carriers from traps that are unstable. Shi et al. [32] discuss the modifications in the electronic structure of luminescent materials that can facilitate the easier release of charge carriers, thus contributing to fading. The reduction in stability of the traps over time can lead to a decrease in luminescent intensity, which is echoed in work by Huntley and Lamothe [33]. Additionally, Clercq and Poelman [34] emphasize the temperature dependence of trapping and detrapping phenomena. They propose that local variations in temperature can lead to differential mobilities in charge carriers, resulting in varying rates of fading depending on environmental conditions. This suggests that temperature management is critical in applications involving TL materials.

In this experiment, the crystal samples were exposed to beta radiation (72 Gy), then stored for various time intervals to observe the reduction in TL signal intensity. The goal was to assess how much energy is lost as a function of time and compare the fading behavior between different samples.

**FIGURE 10:**
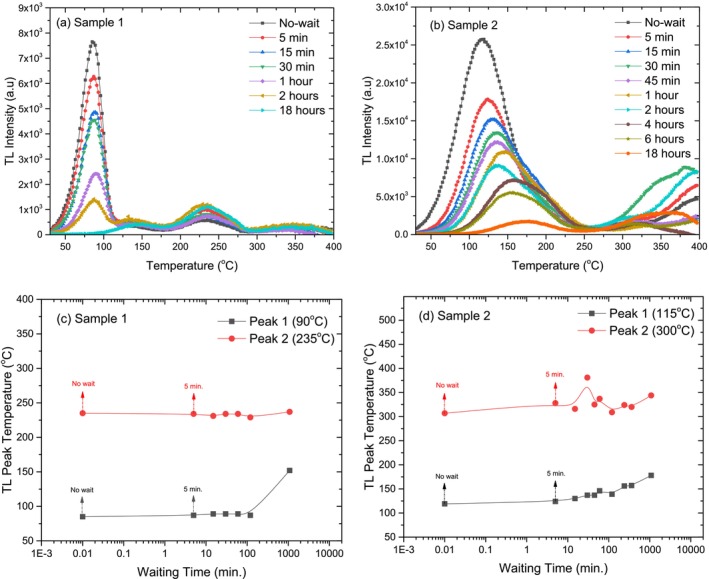
Effects of different waiting times on TL glow curves and TL peak temperatures for Crystal Sample 1 (a, c) and Crystal Sample 2 (b, d).

The TL glow curves and TL peak temperatures for both Crystal Samples as a function of different waiting times are shown in Figure 10. These curves illustrate how the TL glow curve and TL peak temperatures changes over varying intervals between irradiation and reading.

Figure 10a,b shows that both crystal samples exhibit significant TL fading over time, with TL intensity decreasing as the waiting period after irradiation increases. Crystal Sample 1 retains higher TL intensities compared to the Sample 2, but both follow a similar trend of gradual signal loss. Despite this fading effect, the peak temperature remains constant at approximately 100°C, indicating that the energy release mechanism is unaffected. These findings highlight the importance of accounting for fading in TL measurements while confirming that no structural changes occur in the crystals.

The TL peak temperatures for both crystal samples as a function of waiting are presented in Figure 10c,d. It was observed that the TL peak temperature of the high temperature peaks (235°C and 300°C) for both crystals was almost unchanged at different waiting times. However, while the TL peak temperature of the low temperature peak (115°C) for crystals 1 rises 60°C, there appears to be a large shift in the TL peak temperature of the low temperature peak (90°C) for Crystal Sample 2 between two and 18 h waiting time.

**FIGURE 11:**
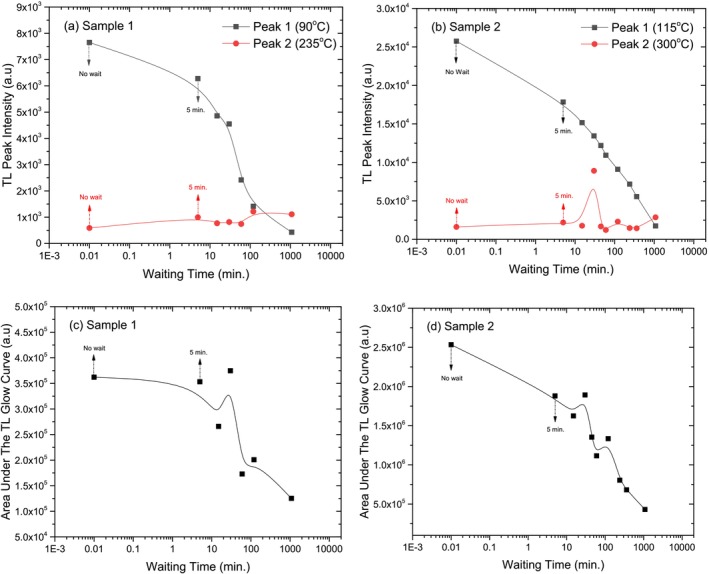
Variations of TL peak intensities area under the TL glow curves as a function of waiting times for Crystal Sample 1 (a, c) and Crystal Sample 2 (b, d).

The changes in the TL peak intensities and area under the TL glow curves for both the crystal samples as a function of waiting time are presented in Figure 11. Figure 11a,b illustrates how the TL peak intensities at different waiting times are affected. For Crystal Sample 1, two distinct peaks are observed: Peak 1 at 90°C and Peak 2 at 235°C. Peak 1 shows a sharp decline (22%) in TL peak intensity in the first 5 min of waiting time and Peak 1 is completely faded after 18 h of waiting time. The TL peak intensity of the peak 2 at 235°C does not seem to be affected during the waiting time.

As in Crystal Sample 1, the TL peak intensities in Crystal Sample 2 show the same behavior for different waiting times. Peak 1 at 115°C shows a sharp decline (33%) in TL peak intensity in the first 5 min of waiting time and it fades about 90% after 18 h of waiting time. No important change in the TL peak intensity of Peak 2 at 300°C is observed as the waiting time increases. Both crystal samples exhibit a clear decline in TL peak intensity at lower temperatures as the waiting time increases. However, in both samples, the higher‐temperature peaks (Peak 2) remain relatively stable, suggesting that the fading effect is more prominent at lower temperatures. Fading refers to the gradual reduction or loss of radiation‐induced signals over time in a sample. This phenomenon can be attributed to various factors, but thermal energy plays a significant role. In thermal fading, lower‐energy traps tend to release their stored energy more quickly because of the higher likelihood of transitions, leading to faster fading. As a result, this can cause substantial errors in dose evaluations [35].

The effects of waiting time on the area under the TL glow curve for both Crystal Samples are presented in Figure 11c,d. For both crystal samples, the graphs show a fluctuating decrease in the area under the TL glow curve as the fading time increases. After 18 h of waiting time, the area under the curve decreased by 65% for Crystal Sample 1 and 88% for Crystal Sample 2. This consistent decline suggests that the energy traps in both crystals are emptied more rapidly over time, indicating a more pronounced fading effect.

Overall, both crystals experience energy loss over time. If these two crystals are compared in terms of the fading, Crystal Sample 2 is more quickly faded than Crystal Sample 1.

## 4 Conclusions

This study comprehensively examined the TL behavior of naturally grown CaCO₃ crystals collected from the Akçatekir region in Adana, Turkey. Different experimental approaches, including dose response, heating rate, reusability, and fading experiments, were used to investigate the factors influencing the TL properties of the crystals. The study evaluated the energy storage, release, and stability characteristics of the crystals under various conditions.

The dose–response experiment shows that both crystal samples exhibit distinct TL glow curves with stable shapes across different radiation doses. TL intensity increases linearly up to 576 Gy before reaching saturation, and higher doses result in a peak shift to higher temperatures before stabilizing. Crystal Sample 1 demonstrates greater stability and consistency compared to Crystal Sample 2. The heating rate experiment shows that increasing the heating rate raises the TL peak temperature while reducing TL intensity for both crystals. Crystal Sample 2 experiences a more significant intensity decrease beyond 4°C/s. However, the TL glow curve shape remains unchanged, indicating no new peaks are formed or lost. Chen's peak shape theory and kinetic models of TL [36] explains that as the heating rate increases, charge carriers are released more quickly, and the system has less time to emit light at each trap depth. This causes the peak temperature to shift because higher temperatures are needed to thermally stimulate the same traps in a shorter amount of time. Additionally, the reduced intensity is due to fewer recombination events occurring at each temperature increment. The reusability experiment confirms that both crystal samples maintain stable TL glow curve shapes over repeated cycles. While low‐temperature peak intensity decreases, the dosimetric peaks near 250°C remain unchanged, indicating stable deeper energy traps. The standard deviation in reusability for Crystal Sample 1 was calculated the ± 2.71%. This stability highlights the crystals' potential for reliable TL applications, such as radiation monitoring. The fading experiment confirms that both crystal samples experience TL signal fading over time as trapped charges gradually dissipate. However, their structural integrity and TL peak positions remain unchanged. Crystal Sample 2 fades more quickly than Crystal Sample 1, indicating a lower capacity for long‐term energy retention.

In conclusion, the naturally grown crystals collected from the Akçatekir region in Adana, Turkey shows good TL properties with clear TL glow curves. They have two distinct TL peaks around 100°C and 250°C. The findings highlight the need for further research to optimize TL materials for specific uses. To increase the dosimetric properties and sensitivity of these crystals, the material can be doped with different foreign atoms. The crystals used in this study can be shown as a good dosimetry candidate in terms of wide linear dose–response, good reusability and the dosimetric peak is not affected by ambient conditions.

## Conflicts of Interest

The authors declare no conflicts of interest.

## Data Availability

The data that support the findings of this study are available from the corresponding author upon reasonable request.
